# *Piper aduncum* polyphenols and flavonoids enhance gut health, immune and anti-inflammatory activity and performance indices of broiler chickens

**DOI:** 10.3389/fvets.2025.1597948

**Published:** 2025-05-27

**Authors:** D. M. Paredes-Lopez, R. A. Robles-Huaynate, R. A. Perales-Camacho, C. V. Alania-Santiago, J. P. Diaz-Gonzales, U. Aldava-Pardave

**Affiliations:** ^1^Department of Animal Science, Universidad Nacional Agraria de la Selva, Tingo María, Peru; ^2^Department of Animal and Public Health, Faculty of Veterinary Medicine, Universidad Nacional Mayor de San Marcos, Lima, Peru; ^3^Postgraduate School, Universidad Nacional Agraria de la Selva, Tingo María, Peru; ^4^Postgraduate School, Universidad Nacional Agraria La Molina, Lima, Peru

**Keywords:** gut health, histomorphometry, microbiota, *Piper aduncum*, polyphenols

## Abstract

High-level use of antibiotics as grow promotors in animal nutrition in the last six decades has pushed to bacterial resistance to these molecules. The search for alternative ways including plants extracts, essential oils or phytochemicals to tackle this problem is increasing nowadays. This study aimed to evaluate the effects of *Piper aduncum* polyphenols (PaP) and flavonoids (PaF) on broiler chicken gut health. 396 Cobb 500 broiler chickens aged 1–33 d old were fed a base diet (BD). Birds were randomly divided into two control (C) and four supplementations (S) groups. C1 was fed with BD and C2 with BD + 50 ppm zinc bacitracin. S1 and S2 were supplemented with 17.5 and 35.0 ppm PaP, whereas S3 and S4 were supplemented with 17.5 and 35.0 ppm PaF of the diet, respectively and sub ministered in drinking water from 1–21 d of age. The *in vivo* gut microbiota at 21 and 28 d of age, gut villi histomorphometry at 7, 14, and 21 d and performance indices at 7, 21 and 33 d were evaluated. Data was processed using a general factorial arrangement. PaP and PaF supplementation, increased lymphocytes and globulins in chickens at 14 d of age (*p* < 0.05), at the same time erythrocytes, granulocytes, and ALT profiles decreased at 21 d of age (*p* < 0.05). *Escherichia coli* and *Staphylococcus aureus* abundance (log_10_ CFU/g) decreased, *Lactobacillus* sp. was enhanced in ileal mucosa and content of chickens at 21 d old on supplementation 35.0 ppm PaP, 17.5 and 35.0 ppm of PaF (*p* < 0.05) and villi length increased with the age of chickens supplemented 17.5 ppm of PaP, 17.5 and 35.0 ppm of PaF (*p* < 0.05). As a result, PaP and PaF maintain weight gain and feed conversion rate, reduce feed intake and improve carcass yield overall in the three stages of broiler chickens. In conclusion, PaP and PaF enhanced gut health, the immune and anti-inflammatory activity, and performance indices of broiler chickens.

## 1 Introduction

Residues from antibiotics, because of indiscriminate use in food animals mainly as growth promotors represent a risk in processing and profitability of livestock production. This adds antimicrobial resistance to certain microorganisms (1, 2), deterioration of the environment, and consequently, deterioration of public well-being and health (1, 3–6). This direct risk to public health has been documented in terms of cancerous properties, modification of microbiota, and potential allergies (7–11).These situations warrant the need to supply innocuous products for food and medication, guaranteeing animal and public health (12–15).

Enzymes, probiotics, prebiotics, herbs, amino acids, immunostimulants, organic acids, bacteriocins, and phytotherapeutic plants have been investigated as antibiotics growth promotors’ alternatives (16–21). These are the most common feed additives that acquire popularity in the poultry industry following the ban of antibiotic growth promoters (AGPs). They are commonly used worldwide because of their unique properties and positive impact on poultry production.

In the National Health Directorate, a variety of plants have been registered, which have been identified for the treatment of several diseases. These plants are known to have been used since ancestral times. Thus, the understanding of their diverse benefits as nutraceuticals or promoters of wellbeing and health (22, 23) is essential. Among these plants, *Piper aduncum* L. (Matico) contains phytochemical components, including polyphenols, flavonoids, triterpenes, alkaloids, and phenylpropanoids (24, 25). It also possesses antibacterial activity against pathogenic organisms (26). Polyphenols and flavonoids are phenol compounds that are extensively present in nature, which are responsible for the reliable performance of plants and their benefits to human health; they have been recognized in numerous studies (27–29).

One of the benefits of phenol compounds is their antioxidant activity (30–33). This eliminates free radicals generated by biomolecules, such as lipids, proteins, and nucleic acids under stressful conditions; this is the basic mechanism for protection against the development of cardiovascular and degenerative diseases, cancer, and diabetes in humans (29, 34, 35).

Another beneficial effect of polyphenols is their antimicrobial activity, wherein phenolic compounds, such as polyphenols and flavonoids reduce the colonization of bacteria with pathogenic potential in the gastrointestinal tract through diverse mechanisms (36–38).

In previous studies on polyphenols, such as curcumin, resveratrol, and epigallocatechin gallate, in broiler chickens, laying hens, and quails, promising results were obtained in terms of the performance of these birds (39–42).

The aim of the present study was to determine the effect of the polyphenol phytocompounds and flavonoids from *P. aduncum* leaves on the enhancing of gut health, as an alternative to antibiotic use as growth promotor in broiler chickens.

## 2 Materials and methods

### 2.1 *Piper aduncum* polyphenols and flavonoids

To obtain *P. aduncum* polyphenols and flavonoids in this study, first the leaves were collected from the plants, in the Luyando district in the Leoncio Prado province of the Huánuco region in Peru, located at 18 L 3981790, UTM 8973000 and 715 m.a.s.l. During the morning hours, the intermediate leaves were collected, carefully cleaned with distilled water and later dried in a stove at 40°C for 72 h. The leaves were ground in a Thomas Willey model 4 brand grinder (United States) until a thick powder was obtained. They were then sifted through a sieve to make the particle size homogeneous. Subsequently, the extract was obtained using the ethanolic extraction method, as described previously (43). This was followed by phytochemical shifting and division of the polyphenols using the Singleton and Rossi (44) colorimetric method and flavonoids according to the method of Kumazawa et al. (45).

### 2.2 Experimental design and broiler chickens

This study was reviewed and approved by the Ethics and Animal Wellbeing committee of the Veterinary Medicine School at the Universidad Nacional Mayor de San Marcos, with authorization, N° 2012–5. The location of this study was at 09° 17′58″ south latitude and 76° 01′07″ west longitude, at a height of 660 m.a.s.l., with an annual precipitation of 3,293 mm, an average annual temperature of 24.85°C, and a relative humidity of 80% (46).

Three hundred sixty Cobb 500 chickens aged 1 day, 40.0 ± 4.0 g weight, were used. The chickens were divided into six groups: C1, C2, S1, S2, S3, and S4, each with five replicates and 12 chickens per each. These birds received similar handling conditions and feeding, where a base diet (BD) was provided during the initial (1–7 d), growth (8–21 d), and finishing phase (22–33 d old). The chickens from C1 were fed with BD, those in C2 with BD + 50 ppm of zinc bacitracin (ZB) (Albac, Norway) in the diet, those in S1 and S2 were fed BD + 17.5 and 35 ppm of PaP, and those in S3 and S4 were fed BD + 17.5 and 35 ppm PaF of the diet via drinking water, from day 1 to 21 d of age. The PaP and PaF were formulated at a concentration of 10 mg/mL in a physiological serum dilutant. At this concentration, the solutions were separated into aliquots at the beginning of the experiment, according to the calculations at 17.5 ppm and 35 ppm of the chickens’ diet obtained for each day of the experiment. The aliquots were refrigerated at 4° C, to allow removal out daily for the volume that corresponded to each day for the S1-S4 groups of experimental chickens.

### 2.3 Experimental diet and nutrition

The diet was formulated in the Mixit-2 program, based on the tables by Rostagno et al. (47). For the preparation, a pre-mix of the micronutrients was made with raw insoluble fiber to obtain good homogenization for the feed. The final mix of the ingredients was made with a horizontal mixer with a capacity of 100 kg, for 10 min. Proximal analysis of the formulated experimental rations was performed for male broiler chickens during the initial, growth, and finishing phases (1–33 d of age). They were in line with the requirements for each phase (Table 1). The nutritional compositions of the initial, growth, and finishing stages were determined based on the requirements of each stage. With this goal, samples of the base diet and the diet supplemented with ZB were analyzed for proximal chemical composition in the Animal Nutrition Laboratory at the Universidad Nacional Agraria de la Selva.

**Table 1:** Diets formulated for male broiler chickens during the initial phase (1–7 d of age), growth (8–21 d of age), and finishing (22–33 d of age).

Inputs (%)	Initial			Growth			Finishing		
	C1	C2	S1–S4	C1	C2	S1–S4	C1	C2	S1–S4
Corn	51	51	51	51.2	51.2	51.2	53.96	53.96	53.96
Palm Oil	4.46	4.46	4.46	4.46	4.46	4.46	5.5	5.5	5.5
Soybean Cake (46%)	39.71	39.72	39.72	39.9	39.9	39.88	36.37	36.37	36.37
Calcium Carbonate	0.89	0.89	0.89	0.79	0.79	0.79	0.75	0.75	0.75
Dicalcium Phosphate	1.81	1.8	1.8	1.8	1.8	1.8	1.58	1.58	1.58
Salt	0.23	0.23	0.23	0.22	0.22	0.22	0.2	0.2	0.2
Pre-mix Vit + Min.	0.15	0.15	0.15	0.15	0.15	0.15	0.1	0.1	0.1
Aflaban	0.05	0.05	0.05	0.05	0.05	0.05	0.05	0.05	0.05
Coccidiostat	0.05	0.05	0.05	0.05	0.05	0.05	0.05	0.05	0.05
Butilated Hydroxytoluene	0.05	0.05	0.05	0.05	0.05	0.05	0.05	0.05	0.05
Choline Chloride	0.25	0.25	0.25	0.2	0.2	0.2	0.2	0.2	0.2
Sodium Butyrate	0.1	0.1	0.1	0.1	0.1	0.1	0.1	0.1	0.1
Sodium Bicarbonate	0.46	0.45	0.46	0.45	0.45	0.45	0.44	0.44	0.44
Lysine (78.4%)	0.34	0.34	0.34	0.22	0.22	0.22	0.24	0.24	0.24
Methionine (99%)	0.25	0.25	0.25	0.23	0.23	0.23	0.22	0.22	0.22
Threonine (98%)	0.11	0.11	0.11	0.09	0.09	0.09	0.09	0.09	0.09
Valine (99%)	0.09	0.09	0.09	0.06	0.06	0.06	0.06	0.06	0.06
BMD (10%)	0	0.01	0	0	0.01	0	0	0.05*	0
Total	100	100	100	100	100	100	100	100	100

C1: Negative control; C2: Positive control; S1: 17.5 ppm of PaP, S2: 35.0 ppm of PaP, S3: 17.5 ppm of PaF, S4: 35.0 ppm PaF of the diet.

### 2.4 Proximal chemical composition of diets

To determine dry matter (DM) content, the samples were dried in an air forced oven (Memmert, UN110 Plus, Germany) at 105°C for 4 h. The samples were analyzed for ashes after 12 h of combustion in a muffle furnace at 600°C (Linn Electro Therm, LM-312.06, Germany). Crude protein (CP) using a Kjeldahl Nitrogen Analyzer (Buchi Digestion Automat, K-438, and Buchi Distillation Unit K-350, Switzerland); Ether extract using an extractor (Aknom XT10, United States). Total fiber was determined by a semiautomatic fiber analyzer (Aknom 200, USA). The nitrogen- free extract was computed as the difference between DM and nutrients determined in the proximal analysis of the diets. The chemical analysis of diets is depicted in Table 2. The free extraction of nitrogen was determined from the difference between DM and nutrients from the proximal chemical analysis.

**Table 2:** Proximal analysis composition of diets for male broiler chickens during the initial, growth, and fattening stages (1–33 days of age).

Diet	Components (%DM)						
	Supplements	Dry matter	Ash	Raw protein	Ethereal extract	Total fiber	NFE
Initial base	C1, S1, S2, S3, S4	90.10	7.12	23.5	5.23	2.43	52. 27
Initial with ZB	C2	90.05	7.10	23,15	5.19	2.49	52.12
Growth base	C1, S1, S2, S3, S4	91.24	6.81	22.13	7.02	2.32	52.96
Growth with ZB	C2	91.52	6.86	22.04	7.16	2.35	53.11
Fattening Base	C1, S1, S2, S3, S4	88.72	6.12	20.34	7.92	2.40	51.91
Fattening with ZB	C2	89.05	6.11	20.41	7.91	2.46	52.14

ZB, Zinc bacitracin at 50 ppm in the diet; NFE, Nitrogen free extract; DM, dry matter. C1: base diet; C2: base diet + 50 ppm de zinc bacitracin, S1: 17.5 ppm of PaP, S2: 35.0 ppm of PaP, S3: 17.5 ppm of PaF, S4: 35.0 ppm of PaF of the diet.

### 2.5 Blood samples and hematological profiles

Blood samples were collected by puncturing the alar veins. Blood for the hematological profiles was obtained in 2-mL vacutainers that contained 2 mg of heparin. The blood for the blood metabolite profiles was obtained in 4-mL vacutainers, which were centrifuged at 1500 rpm for 5 min after coagulation. The serum was separated into 2-mL Eppendorf tubes and stored at −10°C until the analysis with a spectrophotometer. Samples were collected from 30 chickens, 5 chickens per each group of supplementation and controls at 7, 14, and 21 d of age.

The erythrocyte, total and differential leukocyte counts, hematocrit and hemoglobin were determined from whole blood. For the hematocrit, the microhematocrit method and hemoglobin levels, the cyanmethemoglobin method were carried out. Mean corpuscular volume (MCV), mean corpuscular hemoglobin (MCH), and mean corpuscular hemoglobin concentration (MCHC) were also obtained (48).

The glucose level was determined from the blood serum using the glucosidase-peroxidase method. Total protein was obtained using the EDTA/Cu complex in sodium hydroxide. Albumin level was determined using tetrabrom-cresolsulfonphthalein (49, 50). Total cholesterol, alanine aminotransferase, aspartate aminotransferase, total bilirubin, and conjugated bilirubin (Laboratories QAC, España) levels were determined. Measurements were performed at 515 and 530 nm using an Auto Chemistry Analyzer-AS 830 spectrophotometers (Italy).

### 2.6 Intestinal content and microbial culture

Four chickens from each of the six supplementation groups were chosen at random at 21 and 28 days of age and sacrificed by atlanto-occipital dislocation. Their digestive tracts were immediately dissected and a segment of approximately 30 cm of the ileum, beside Meckel’s diverticulum, toward the blind (51, 52), was opened to obtain the intestinal content. This included scraping the mucosa and collecting it on sterile petri dishes.

Native species from the microbiota of broiler chickens, such as *Escherichia coli*, *Lactobacillus* sp., and *Staphylococcus* sp., were grown (51). For the cultivation of *E. coli*, MacConkey agar; for *Lactobacillus* sp., MRS agar, and for *S. aureus*, mannitol salt agar was used (Merck, Darmstadt, Germany). The culture petri dishes were incubated for 24 h at 37°C. The bacterial count was expressed as the base 10 logarithm of the number of colony-forming units per gram (log_10_CFU/g) of ileal content (51, 52).

### 2.7 Intestinal tissue and histomorphometry

Five chickens from each of the six treatments were randomly selected at 14, 21, and 28 d of age and sacrificed by atlanto-occipital dislocation. Their digestive tracts were immediately dissected. A segment approximately 5 cm from the middle part of the duodenum, jejunum, and ileum segments (51, 52) was taken, opened lengthwise, and transversely sectioned. They were removed and submerged three to four times in a sterile physiological serum solution to remove the intestinal content from the mucus. They were later attached with staples to sturdy posterboard bases to keep the segments straight. The three segments from each bird were stored in 100 mL of 10% formalin solution with physiological serum. The stored intestinal samples were processed using conventional histological methods and stained with hematoxylin and eosin (53).

Measurements of the length and width of the intestinal villi and depth of the crypts were taken by measuring 10 villi at 10X. The average of each intestinal segment, corresponding to each animal, was determined. A Leica® DM500 optical microscope and LAS EZ Leica® program, installed on a computer connected to the Leica® microscope were used. This also included a Leica® ICC50 camera. This system allows the distance between any pair of points chosen by the user to be determined. The villus length was measured from its apex to the apex of the Lieberkühn crypt entrance. The width of the villi was measured as the line perpendicular to the middle section of the villi. The depth of the Lieberkühn crypt was measured from the entrance to the crypt to its base zone. The measurements were recorded in micrometers.

### 2.8 Performance indices

At 7, 21, and 28 d of age, all chickens were weighed and feed consumption was recorded, and the following indices were determined:

- Feed intake (FI) was computed from the relationship between the total consumption of the lot and the number of birds in the lot.
- Weight gain (WG) was computed using the relationship between the final weight minus initial weight of the lot and number of birds in the lot.
- Feed conversion rate (FCR) was computed from the relationship between feed consumption and weight gain.
- Carcass yield: Was obtained from the relationship between weigh of carcass without any disposal and chicken live weight at 33 d old.

### 2.9 Statistical analyses

To evaluate the effect of PaP and PaF supplementation on the variables under study in relation to the age of the chicken, the data on blood metabolite profiles, measures of the villi, and Lieberkühn crypts in the intestinal segments were processed using a general factorial arrangement with three ages of chickens, two PaP and PaF levels + two controls, and for bacterial count, two ages of chickens were considered. The guide for statistical analysis was obtained from Pollesel et al. (54) and Bashir et al. (55). The data for the height and width of the villi, depth of the crypts, microbial abundance, hematological data, and the performance indices were primarily transformed using the square root, Box-Cox, or base 10 algorithms. Normality and homogeneity were later proven using the Shapiro–Wilk’s and Levene’s tests, respectively. A two tailed analysis of variance (ANOVA) was used to analyze the averages of the blood metabolites and hematological profiles, abundance of microbiota, and measurements of the villi and crypts. Tukey’s test was used to establish their significance (*p* ≤ 0.05). The Software Statistics Infostat (56) was used to process the data. The data for the productive indices were subjected to a completely randomized design, and the averages were compared using the student–Newman–Keuls (SNK) test (5%).

## 3 Results

### 3.1 Quantification of phenol compounds and flavonoids

In this study, in the *P. aduncum* leaves in a dry base, 12.5 mg of GAE/g of dry ethanol extract from phenol compounds and 0.2 mg of QE/g of dry extract from flavonoids were obtained. In this study, the mayor active components into these two groups of *Piper aduncum* phytocompounds have not being isolated. However, in previous studies dillapiod, asaricin, elemicin and myristicine as propenylphenols, pinostrobin and sakuranetin as flavonoids components have been identified in *Piper aduncum* (25, 57).

### 3.2 Hematology and blood metabolites profiles

The hematocrit, hemoglobin, and total erythrocyte profiles; MCV, MCH, and MCHC indices; and total leukocyte, lymphocyte, and granulocyte profiles of broiler chickens supplemented with PaP and PaF are depicted in Table 3. The hematocrit, hemoglobin, and total erythrocyte profiles, as well as the MCV and MCH indices, decreased in chickens supplemented with 17.5 and 35.0 ppm of PaP and 50 ppm of ZB, in comparison to those obtained for the C1 group chickens at 14 d (*p* < 0.05). The lymphocytes increased in the chickens because of supplementation with 35 ppm PaP, 17.5 and 35.0 ppm PaF, and C2 (*p* < 0.05) at 14 d of age (Table 3). Conversely, the granulocytes decreased in the chickens that were given supplements of 35 ppm PaP, 17.5, and 35 ppm PaF in the C2 group, in comparison to those in the C1 group at 14 d of age (*p* < 0.05).

**Table 3:** Hematological profiles of broiler chickens supplemented with *Piper aduncum* polyphenols and flavonoids.

Variables	Hematological profiles and indices									
	Hto (%)		Hb (g/dL)	Ery(x10^6^/mm^3^)	*Leu(x10^3^ mm^3^)	Lyn(%)	Gra (%)	MCV (fL)	MCH (pg)	MCHC (gdL^−1^)
Age (A)	0.0000		0.0000	0.0000	0.0000	0.0000	0.0000	0.0000	0.0000	0.0005
Supplementation (S)	0.1933		0.1760	0.1941	0.0191	0.0010	0.9679	0.1529	0.0040	0.6896
A x S	0.0001		0.0001	0.0002	0.1185	0.0000	0.0000	0.0000	0.0000	0.0847
VC	10.66		10.46	9.33	8.59	8.88	12.17	1.39	1.19	0.4106
R^2^adjusted	72.04		72.42	71.94	38.84	96.55	95.78	70.21	74.50	17.52
Age (days)										
7	23.50c		7.80c	2.75c	10.82b	18.70c	79.56a	85.39c	28.39c	33.20a
14	25.88b		8.61b	2.98b	8.98c	69.88b	27.59b	86.54b	28.75b	33.20a
21	33.70a		11.15a	3.77a	12.72a	73.33a	25.00b	89.32a	29.55c	33.08b
Supplementation										
C1	28.93		9.61	3.29	11.28ab	52.53b	44.73	87.68	29.14a	33.18
C2	27.07		8.97	3.11	9.46b	55.0ab	43.47	86.69	28.72bc	33.13
S3	26.82		8.91	3.08	11.05ab	52.6b	45.24	86.88	28.80bc	33.15
S4	28.00		9.28	3.20	11.25ab	54.6ab	43.41	87.18	28.97ab	33.16
S5	26.53		8.79	3.05	11.88a	57.80a	41.73	86.61	28.64c	33.13
S6	29.00		9.62	3.30	9.85b	52.94b	44.33	87.55	29.12a	33.19
Supplementation by Age										
Supplementation	Age (days)									
C1	7	20.40	6.78	2.44	10.42	20.60	78.00	84.14	27.98b	33.23
C2	7	25.20	8.38	2.92	10.29	17.80	80.40	86.24	28.68a	33.25
S1	7	24.00	7.94	2.80	12.75	19.20	78.80	85.54	28.30ab	33.08
S2	7	24.17	8.03	2.82	10.85	18.83	79.50	85.70	28.65a	33.24
S3	7	23.60	7.82	2.76	10.95	18.20	79.80	85.35	28.12ab	33.13
S4	7	23.50	7.82	2.75	10.02	17.67	80.67	85.36	28.56ab	33.26
C1	14	31.20a	10.40a	3.50a	9.99	59.40b	35.40a	89.13a	29.69a	33.17
C2	14	21.40c	7.08c	2.504c	6.55	75.80a	22.80b	84.22c	27.86c	33.08
S1	14	23.5bc	7.88bc	2.75bc	8.91	60.00b	37.17a	85.9bc	28.64b	33.34
S2	14	25.8bc	8.56bc	2.98bc	10.72	74.80a	23.40b	86.4bc	28.66b	33.18
S3	14	24.6bc	8.16bc	2.86bc	10.42	76.60a	22.40b	85.9bc	28.50b	33.17
S4	14	28.7ab	9.52ab	3.27ab	8.18	73.83a	23.33b	87.6ab	29.10a	33.20
C1	21	35.20	11.66	3.92	13.78	77.60a	20.80	89.76	29.74	33.13
C2	21	34.60	11.44	3.86	12.57	71.4ab	27.20	89.61	29.62	33.06
S1	21	32.50	10.73	3.65	12.18	73.0ab	25.33	88.99	29.39	33.03
S2	21	33.67	11.13	3.77	12.14	73.5ab	24.00	89.34	29.54	33.07
S3	21	31.40	10.38	3.54	14.70	78.60a	23.00	88.57	29.29	33.07
S4	21	34.83	11.53	3.88	11.65	67.33b	29.00	89.65	29.69	33.12

^abc^Different letters in each line denote differences between supplementations in the SNK test (*p* < 0.05). C1: base diet; C2: base diet + 50 ppm de zinc bacitracin, S1: 17.5 ppm of PaP, S2: 35.0 ppm of PaP, S3: 17.5 ppm of PaF, S4: 35.0 ppm of PaF of the diet. Hto: hematocrit, Hb: hemoglobin, Ery, Erythrocytes; Leu, leukocytes; Lyn, Lymphocytes; Gra, granulocytes; VC, variation coefficient; *Transformed by a natural logarithm.

The glucose, cholesterol, triglyceride, aspartate transaminase, protein, alanine transaminase, albumin, and globulin levels in broiler chickens are depicted in Table 4. At 14 d of age, the globulin profiles increased in the chickens supplemented with 17.5 and 35.0 ppm of PaF, in comparison to the obtained for those supplemented with PaP and the chickens from groups C1 and C2 (*p* < 0.05; Table 4). Conversely, the albumin profile of the chickens supplemented both levels of PaF decreased, in comparison to that obtained from the chickens in the groups supplemented PaP, C1, and C2 (*p* < 0.05; Table 4). Similarly, the triglyceride profile decreased in the chickens supplemented with the two levels of PaP and PaF and with C2, at 14 d of age (*p* < 0.05; Table 4) and at 21 d of age the ALT profiles also decreased in the chickens supplemented with 17.5 PaP and the two levels of PaF (*p* < 0.05; Table 4).

**Table 4:** Variance analysis of blood metabolites profiles of broiler chickens supplemented with *P. aduncum* polyphenols and flavonoids.

Variables		Blood metabolites							
		Glu(mg)	*Cho (mg)	*Trig(mg)	*AST (IUL^−1^)	Pro(gdL^−1^)	*ALT (UIL^−1^)	**Alb (gdL^−1^)	Glo (gdL^−1^)
Age (A)		0.0000	0.0000	0.0077	0.1012	0.0000	0.0000	0.0000	0.0000
Supplementation (S)		0.0681	0.0787	0.0000	0.2507	0.0150	0.0076	0.0011	0.0011
A x S		0.0012	0.5428	0.0011	0.0646	0.1243	0.0035	0.0000	0.0000
VC		14.56	2.90	5.74	2.91	11.36	7.83	6.87	19.16
R^2^ adjusted		61.03	48.91	38.12	11.82	27.10	44.18	65.88	62.54
Ages (days)									
7		211.94a	138.58a	56.63ab	214.89	2.34b	12.52c	1.27c	1.07a
14		198.73b	143.65a	60.98a	226.33	2.58a	17.96a	1.83a	0.69b
21		142.24c	106.11b	50.84b	233.82	2.67a	15.77b	1.53b	1.13a
Supplementation									
C1		179.80	128.69	70.42a	221.47	2.57a	17.11a	1.50bc	0.99b
C2		181.00	122.72	52.99bc	215.50	2.41ab	15.7ab	1.53abc	0.87b
S1		197.00	124.09	51.95bc	214.35	2.63a	13.22b	1.70a	0.89b
S2		171.00	138.18	61.36ab	234.32	2.60a	16.69a	1.61ab	0.96b
S3		183.53	122.32	45.42c	240.32	2.32b	14.92ab	1.41c	0.92b
S4		190.88	132.38	56.7b	225.41	2.61a	14.64ab	1.46bc	1.13a
Supplementation by Age									
Supplementation.	Age (Days)								
C1	7	212.80	139.25	53.43	206.05	2.30	11.98	1.22	1.08
C2	7	189.20	132.48	52.73	199.80	2.22	10.74	1.15	1.06
S1	7	223.00	127.30	55.69	211.95	2.40	10.94	1.29	1.10
S2	7	189.83	166.21	64.89	212.44	2.38	13.74	1.26	1.12
S3	7	223.00	132.18	44.38	220.56	2.22	15.01	1.26	0.96
S4	7	236.00	134.65	71.26	242.06	2.52	13.40	1.46	1.06
C1	14	176.20bc	141.03	110.59a	205.19	2.76	18.19	2.01a	0.58b
C2	14	213.40abc	139.47	58.5b	220.64	2.44	21.98	1.93a	0.50b
S1	14	229.50a	138.74	49.53b	239.60	2.82	15.45	2.26a	0.53b
S2	14	165.33c	147.53	68.25b	246.19	2.73	19.26	2.10a	0.62b
S3	14	188.00abc	139.03	45.14b	241.71	2.32	17.21	1.38b	0.98a
S4	14	216.83ab	154.85	54.32b	206.25	2.35	16.86	1.40b	0.94a
C1	21	150.40	108.51	59.10	256.94	2.64	22.97a	1.34	1.30a
C2	21	140.40	100.04	48.23	227.02	2.58	16.38ab	1.57	1.06ab
S1	21	138.50	108.18	50.82	193.93	2.68	13.68b	1.63	1.05ab
S2	21	157.83	107.59	52.19	246.00	2.68	17.58ab	1.55	1.13ab
S3	21	139.60	99.58	46.76	260.33	2.42	12.86b	1.60	0.82b
S4	21	127.33	111.59	48.95	232.15	2.95	13.67b	1.53	1.39a

^abc^Different letters denote statistic difference, SNK (*p* < 0.05); Cho, Trig, AST, ALT data were transformed with a natural logarithm and Alb data with quadratic root, respectively. Abc: Different letters in lines denote differences, SNK test (*p* < 0.05); VC: variation coefficient, Cho: cholesterol, Tri: triglycerides, Glu: glucose, Pro: protein, Alb: albumin, Glo: globulin, AST: aspartate transaminase, ALT: alanine transaminase; C1: base diet; C2: base diet + 50 ppm de zinc bacitracin; S1: 17.5 ppm of PaP, S2: 35.0 ppm of PaP, S3: 17.5 ppm of PaF, S4: 35.0 ppm of PaF of the diet.

### 3.3 Antimicrobial activity

**Figure 1:**
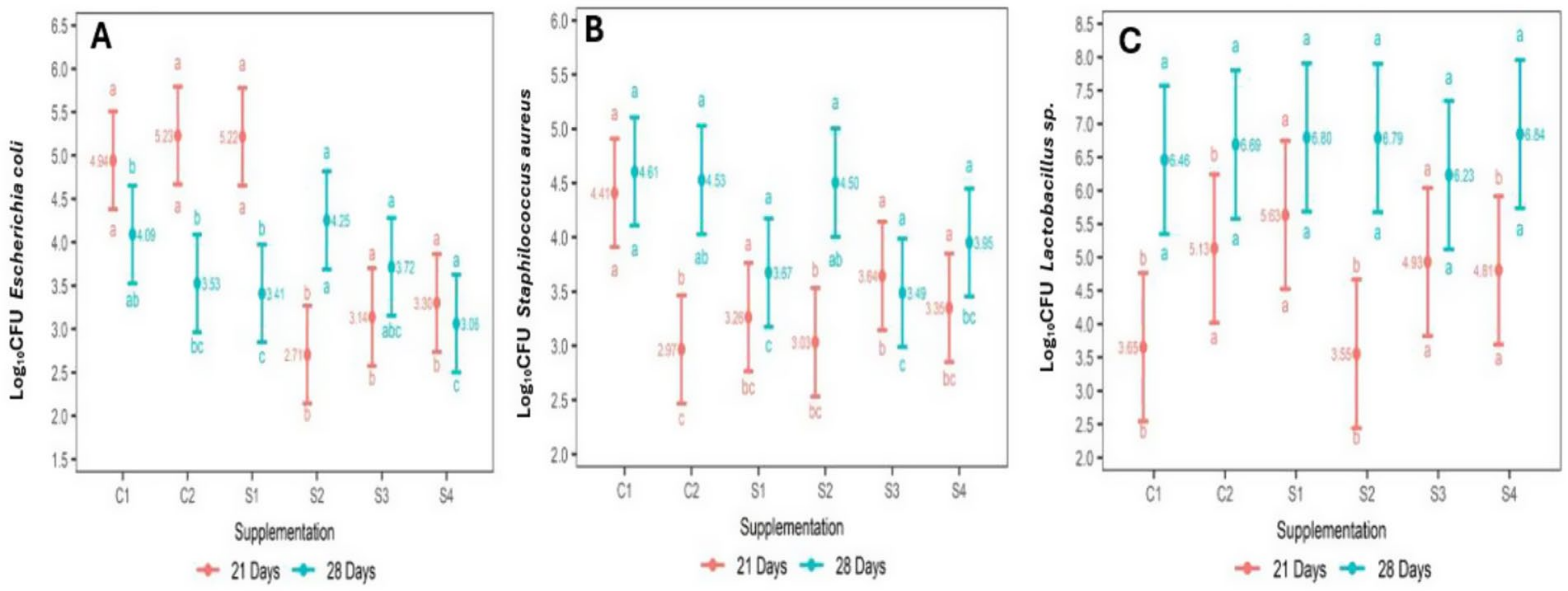
Interaction of PaP and PaF effect and Chickens age on *Escherichia coli* **(A)**, *Staphylococcus aureus* **(B)** and Lactobacillus sp. abundance. **(C)** abc: different letters at the bottom of intervals denotes dependance of supplementations on chicken age, (SNK) test (*p* < 0.05). different letter at the top of intervals denotes dependance of age on PaP and PaF supplementation (SNK) test (*p* < 0.05). CFU: Colony forming units; C1 (−): Base diet, C2 (+): Base diet+ZB, S1: 17.5 ppm PPa, S2: 35 ppm PPa, S3: 17.5FPa, S4: 35 ppm FPa.

The microbial population obtained from the content and mucosa of the broiler chicken ileum at 21 and 28 d of age, as log_10_ CFU/g of fresh intestinal content, is depicted in Table 5 and Figure 1. The abundance (log_10_ CFU/g) of *E. coli* decreased in the groups of chickens that were supplemented with 35 ppm of PaP and 17.5 and 35 ppm of PaF, at 21 d of age, in comparison to the group C1 and C2 (*p* < 0.05; Table 5; Figure 1A). In addition, in the groups of chickens supplemented 17.5 and 35 ppm of PaF, the abundance was like that obtained at 28 d of age (*p* > 0.05; Figure 1A).

**Table 5:** Abundance (log_10_CFU/g) of *Escherichia coli*, *Staphylococcus aureus* and *Lactobacillus* sp. in the ileum mucosa content of broiler chickens supplemented with *P. aduncum* polyphenols and flavonoids.

Variables	Bacterial abundance (log_10_CFU)			
	*Escherichia Coli*	*Lactobacillus* sp.		*Staphilococcus aureus*
Age (A)	0.0003	0.0000		0.0000
Supplementation (S)	0.0000	0.0153		0.0000
A x S	0.0000	0.0262		0.0000
VC	9.12%	12.42%		8.29%
Adjusted R^2^	84.25%	71.84%		76.21%
Age (days)				
21	4.09a	4.62b		3.44b
28	3.68b	6.64a		4.13a
Supplementation				
C1	4.52a	5.06b		4.51a
C2	4.38a	5.91ab		3.75b
S1	4.31a	6.22a		3.47b
S2	3.48b	5.17b		3.77b
S3	3.43b	5.58ab		3.57b
S4	3.18b	5.83ab		3.65b
Supplementation by Age				
Supplementation	Age (days)			
C1	21	4.94a	3.65b	4.41a
C2	21	5.23a	5.13a	2.97c
S1	21	5.22a	5.63a	3.26bc
S2	21	2.71b	3.55b	3.03bc
S3	21	3.14b	4.93a	3.64b
S4	21	3.30b	4.81a	3.35bc
C1	28	4.09ab	6.46	4.6a
C2	28	3.53bc	6.69	4.53ab
S1	28	3.41c	6.80	3.67c
S2	28	4.25a	6.79	4.5ab
S3	28	3.72abc	6.23	3.49c
S4	28	3.06c	6.85	3.95bc

^abc^Different letters denote statistic difference, SNK (*p* < 0.05); VC, variation coefficient; C1: base diet; C2: base diet + 50 ppm de zinc bacitracin; S1 and S2: polyphenols at 17.5 and 35.0 ppm; S3 and S4: flavonoids at 17.5 and 35.0 ppm of the diet. CFU: Colony forming unit.

The abundance (log_10_CFU) of *S. aureus* decreased in all groups of chickens supplemented with PaP and PaF, in comparison to the C1 group (*p* < 0.05) at 21 d of age (Table 5). Similarly, in chickens supplemented with 17.5 ppm PaP, 17.5 and, 35 ppm PaF, this abundance remained at the same level as that of the chickens at 28 d of age (*p* > 0.05; Figure 1B). Conversely, for the broiler chickens supplemented with 17.5 ppm of PaP and both levels of PaF, the abundance of *Lactobacillus* sp. increased in comparison to the C1 group at 21 d of age (*p* < 0.05; Table 5; Figure 1C). Notwithstanding, the abundance (log_10_ CFU) of *Lactobacillus* sp. in all groups of supplemented chickens was similar to that obtained in the C1 group at 28 d of age (*p* > 0.05; Table 5).

### 3.4 Intestinal histomorphometry

**Figure 2:**
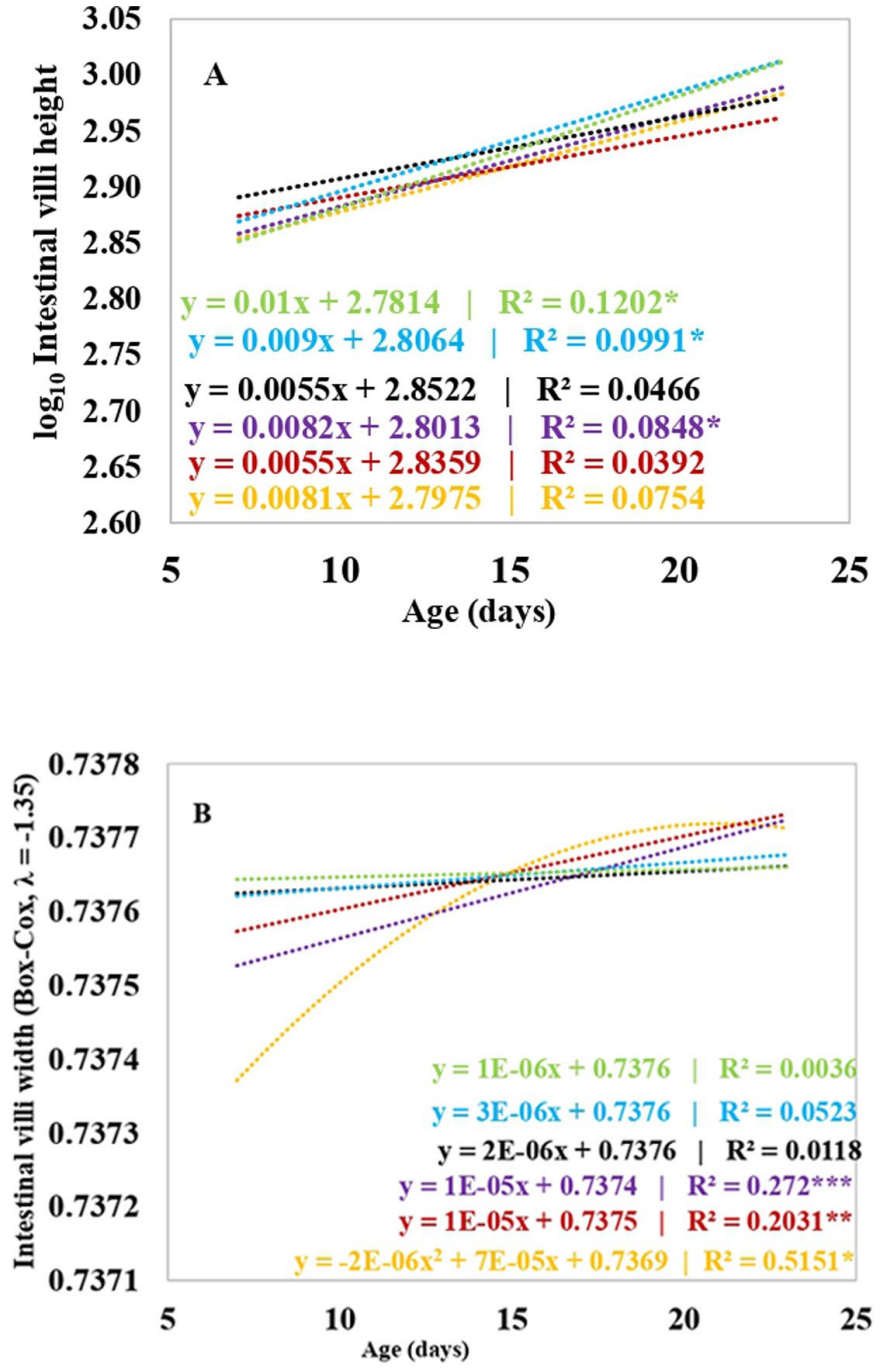
Regression analysis from the effect of the broiler’s chickens age on the villi height **(A)** and villi width **(B)** on supplementation with polyphenols and flavonoids from *Piper aduncum* leaves. *p < 0.05; **p < 0.01; ***p < 0.001. C1 (−): Base diet, C2 (+): Base diet+ZB, S1: 17.5 ppm PaP, S2: 35 ppm PaP, S3: 17.5 ppm PaF, S4: 35 ppm PaF.

Table 6 and Figure 2 show the results of the measurements of the height and width of the duodenum, jejunum and ileum villi, depth of the Lieberkühn glands and the index for the height and depth of the crypts of broiler chickens supplemented with 17.5 and 35 ppm PaP and PaF. The height of the intestinal villi increased linearly with the age of the chickens and was dependent on the age of the broiler chickens under the effect of 17.5 ppm PaP and 17.5 and 35 ppm PaF (*p* < 0.05), compared to that in chickens in groups C1 and C2 (Figure 2A).

**Table 6:** Histomorphometry of the villi from duodenum, jejunum, and ileum of broiler chickens supplemented with *P. aduncum* polyphenols and flavonoids.

Variables		Measurements (μm)			
		Villi height	Crypt Dept	Villi width	VH/CD
Age (A)		0.0000	0.0000	0.0000	0.0000
Intestinal segment (IS)		0.0000	0.0000	0.0000	0.0000
Supplementation (S)		0.2594	0.6015	0.0058	0.5879
A x IS		0.0663	0.0013	0.0001	0.0077
A x S		0.0377	0.0968	0.0000	0.2107
IS x S		0.8695	0.2513	0.5690	0.3906
A x IS x S		0.7108	0.7369	0.3186	0.8031
CV		1.87%	3.14%	0.02%	10.91%
Adjusted R^2^		91.63%	54.22%	37.14%	70.70%
Intestinal segment					
Duodenum		1386.91a	222.50a	121.18a	6.23a
Jejunum		791.68b	180.83b	115.32b	4.38b
Ileum		544.19c	161.09c	114.30b	3.39c
Age (days)					
7		748.55c	175.64b	112.06c	4.26c
14		806.67b	169.14b	117.32b	4.77a
23		990.82a	218.70a	121.59a	4.53b
Supplementation					
C1		823.05	184.09	112.89b	4.47
C2		824.58	182.28	118.18a	4.52
S1		833.42	185.80	116.11a	4.49
S2		857.78	184.29	117.64a	4.65
S3		866.83	189.40	118.07a	4.58
S4		847.78	192.56	118.35a	4.40
Supplementation by Age					
Supplementation	Age (Days)				
C1	7	753.62		100.78c	
C2	7	761.24		111.21ab	
S1	7	727.02		108.59b	
S2	7	790.88		117.48a	
S3	7	751.18		116.56a	
S4	7	713.20		118.73a	
C1	14	751.68		116.84	
C2	14	793.68		120.82	
S1	14	812.43		117.83	
S2	14	825.86		115.53	
S3	14	832.39		117.05	
S4	14	829.06		116.56	
C1	23	1002.25		123.2	
C2	23	927.96		123.33	
S1	23	980.07		123.03	
S2	23	966.30		120.03	
S3	23	1041.68		120.70	
S4	23	1030.52		119.80	

^a–c^Different letters denote statistic difference, SNK test (*p* < 0.05); VH, villi height; CD, crypt depth; VW, villi width. C1: base diet; C2: base diet + 50 ppm de zinc bacitracin; S1 and S2: polyphenols at 17.5 and 35.0 ppm; S3 and S4: flavonoids at 17.5 and 35.0 ppm of the diet. VH and VW data were transformed using base 10 logarithm and CD data with Box-Cox using lambda = −1.353, respectively.

Likewise, the width of the chicken villi increased with age, in a linear tendency, and was dependent on age on supplementation with 17.5 ppm PaP (*p* < 0.001; Table 6 and Figure 2B). Additionally, an increase in the width of the villi in chickens supplemented with 35 ppm PaP and 17.5 and 35 ppm PaF at 7 d of age was observed in comparison to that obtained for the chickens from group C1 (*p* < 0.05; Table 6).

### 3.5 Performance indices

Weight gain, feed conversion rate, and feed intake indices of the broiler chickens were evaluated as the principal indices for evaluating the performance of animal production during each of the stages: initial, growth, and fattening, as well as the total weight from the three phases (58). Table 7 depicts the results of these indices under the effect of supplementation 17.5 and 35 ppm PaP and PaF of the diet and supplemented in drinking water. PaP and PaF maintain weight gain and feed conversion rate (*p* > 0.05), reduce feed intake and improve carcass yield overall in the three stages of broiler chickens in comparison to that obtained for the C1 and C2 chickens’ groups (*p* < 0.05; Table 7).

**Table 7:** Performance indices of broilers chickens supplemented with *P. aduncum* polyphenols and flavonoids.

Performance indices	Chickens stage	ANOVA			Supplementation					
		*p*-value	R^2^ ajusted (%)	VC (%)	Control		Polyphenols (ppm)		Flavonoides (ppm)	
					(C1)	+ (C2)	17.5(S1)	35.0(S2)	17.5(S3)	35.0(S4)
Weight gain (g)	Initial	0.462	0.00	5.84	119.40	121.26	123.15	119.23	120.21	114.75
	Growth	0.14	11.53	5.10	570.75	569.11	550.84	532.23	536.79	524.51
	Fattening	0.0004	38.73	6.03	1140.25a	1135.35a	996.42c	1044.51ab	1112.08a	1021.61b
	Overall	0.0045	28.69	5.51	1808.21a	1825.72a	1670.40ab	1695.97ab	1757.77ab	1635.21b
Feed intake (g)	Initial	0.838	0.00	6.04	148.78	150.17	154.84	148.34	149.79	149.07
	Growth	0.0806	16.09	7.14	907.82	884.82	831.55	851.29	880.84	802.14
	Fattening	0.0132	28.62	6.63	1730.07ab	1777.26a	1565.86b	1569.34b	1697.98ab	1613.56ab
	Overall	0.000	42.54	4.82	2758.45ab	2873.18a	2552.25b	2568.97b	2720.28ab	2560.67b
Feed conversion rate	Initial	0.216	1.45	1.44	1.24	1.24	1.26	1.24	1.26	1.26
	Growth	0.000	51.68	6.72	1.61a	1.55a	1.51a	1.60a	1.64a	1.31b
	Fattening	0.153	10.74	6.59	1.52	1.57	1.57	1.51	1.50	1.54
	Overall	0.759	0.00	4.82	1.52	1.54	1.53	1.52	1.55	1.52
Carcass yield (%) Overall		0.005	17.21	2.61	74.40ab	73.97b	75.98ab	76.59a	76.62a	76.47a

^abc^Different letters denote statistic difference, SNK (*p* < 0.05). VC, variation coefficient.

## 4 Discussion

The objective of this study was to evaluate the effects of polyphenols and flavonoids from *Piper aduncum* on the intestinal health of broiler chickens. The polyphenols and flavonoids comprise phenol compounds that are extensively present in nature and are responsible for the good functioning of plants. Their benefits to human health have been recognized in diverse studies (27–29). Polyphenols have increased weight gain, decreased the feed conversion rate in broiler chickens, an increase in the production and quality of eggs in laying hens and quails (40–42). Previous studies have demonstrated that *Piper aduncum* contains diverse phytochemical compounds, including polyphenols and flavonoids, which possess antibacterial activities (24, 26). Supplementation of chickens with 17.5 and 35 ppm of PaP and FaP in this study increased the lymphocyte, globulin, height and width of the intestinal villi and abundance of *Lactobacillus* sp. However, the triglyceride, ALT, albumin, erythrocyte, MCV, MCH, granulocyte profiles and similarly, the abundance of *E. coli* and *Staphylococcus aureus* were reduced. Furthermore, PaP and PaF improve carcass yield, maintain weight gain and feed conversion rate and reduce feed intake overall in the three stages of broiler chickens. To our knowledge there is not information about whether *Piper aduncum* preparations are registered as feed supplements and are available on the market.

### 4.1 Quantification of phenol compounds and flavonoids

Most studies on the determination of the phytochemical composition of plants have focused on the determination of antimicrobial and antioxidant activities *in vitro*. Herein in this study, *in vivo* antimicrobial activity, changes in histomorphometry of the gut, and in the performance indices of broiler chickens by PaP and PaF were determined.

The *P. aduncum* phytocomponent groups obtained from the phytochemical screening in this study revealed components and gradations like those obtained in previous studies of this plant (59, 60). Nonetheless, the quantification of PaP and PaF obtained in this study has not been performed in previous studies (25, 59, 61, 62).

The total of polyphenols from 12.5 mg of GAE/g of dry ethanol extract and 0.2 mg of QE/g of flavonoids from dry extract proves that the *P. aduncum* leaves are an important source of polyphenols, and flavonoids. However, previous studies of these phytocompounds in other plants, such as *Fragaria chiloensis* spp. leaves have revealed 19.9 mg of GAE/g of dry methanol extract (DME) of polyphenols and 8.3 mg of QE/g of DME of flavonoids (33). In *Murraya koenigii* and *Micromelum*, 101 and 83 mg of GAE/g of dried ethanolic extract (DEE) of polyphenols, and 9.75 and 9.16 mg of QE/g of DEE of flavonoids have been obtained, respectively (63).

In general, the composition and content of polyphenols in plants depend on factors, such as species, genetics (variety and clones), and environmental factors, such as exposure to light, temperature, and soil. This includes farming practices, such as irrigation, nitrogen availability, and diverse management parameters for farming (64).

### 4.2 Hematology and blood metabolites profiles

To our knowledge, no previous studies have been carried out on the effects of supplementation with polyphenols and flavonoids from *P. aduncum* (PaP and PaF) on the hematological and blood metabolites profiles of broiler chickens.

Reduction in erythrocytes, hematocrit, hemoglobin profiles, and the MCV and MCH indices because of PaP, as well as the granulocytes because of PaF effect at 14 d of age in the present study is related to the results of our previous study in which the erythrocytes, hematocrit, hemoglobin and granulocytes profiles, and the MCV and MCH indices decreased and lymphocytes increased in chickens at 28 d old as the ethanolic extract of *P. aduncum* leaves increased from 0, 50 and 100 ppm of the diet. The erythrocytes of birds, like the erythrocytes of fish, are proposed to release cytokines and interferons (65–67), molecules with inflammatory activity that are released only by leukocytes. Therefore, the decrease in the erythrocytes and granulocytes of the experimental chickens at 14 days of age in this study (Table 3) could be associated with the anti-inflammatory activity of PaP through mechanisms that decrease the population of heterophils, erythrocytes and the expression of pro-inflammatory genes. These results could be associated to the fact that within the polyphenols from *P. aduncum* are found phytocomponents that have similar functions as dillapiole which has anti-inflammatory activity, identified in *P. aduncum* (68), and others attributed to *P. umbellaceum* (69), or like curcumin and resveratrol polyphenols. These polyphenols have been proven to have the characteristic of modulating the expression of pro-inflammatory genes, a decrease in heterophils, as well as the production of cytokines in previous studies in chickens (70–75).

In addition to the anti-inflammatory activity of PaP and PaF in this study, an increase in the levels of lymphocytes and globulins in broiler chickens at 14 d of age (Table 3) could also be associated with the immunostimulatory activity of the PaP phytocompounds. Lymphocyte proliferation and immunoglobulin titers are immunological markers that indicate humoral and cellular immune activity (76). Previous studies with other polyphenols, such as coumarin and resveratrol, have also proven to enhance the natural and acquired immunity of chickens and laying hens under stressful conditions because of heat or bacterial infection (71, 72, 77, 78).

Triglycerides are lipids that are synthesized by hepatic tissue and are present at the highest levels in vertebrates, including birds, and its main role is to serve as energy reserve (79). Around 50–70% of the fat in the diet is digested by pancreatic lipase, one of the main lipolytic enzymes, which also converts triglyceride substrata into free fatty acids and triglyceride monoesters (80, 81).

The decrease in blood lipids, such as triglycerides, in chickens supplemented with PaP and PaF at 14 d of age in this study (Table 4; Figure 2B) could be associated with the inhibition of pancreatic lipase by the PaP phytocompounds. These results align with those obtained in a previous study, wherein the ethanolic extract from *P. aduncum* decreased the cholesterol profiles in rats (60) and other studies that have demonstrated that the polyphenols derived from fruits and vegetables inhibit the activity of pancreatic lipase, as well as the cholesterol esterase (82). These results differ from those of a previous study, wherein the triglyceride profiles increased in broiler chickens at 28 d of age under the effect of *P. aduncum* ethanol extract (23). To our knowledge, this is the first study to report the modulation of erythrocyte, granulocyte, lymphocyte, globulin, and triglyceride profiles by PaP and PaF in chickens.

### 4.3 Antimicrobial activity

The chicken microbiota is made up of different phyla of microorganisms where commensal and symbiotic microorganisms and potential pathogens participate (83, 84). The microbial community is regulated by distinct groups of additives, including essential oils and extracts (85, 86). The antimicrobial activity of an extract or essential oil from a plant depends on diverse factors, among which are the chemical structure of its components (87, 88). In this manner, it is primarily attributed to the polyphenol content, and their concentration determines the antimicrobial potential of a plant (36, 89, 90). In this study the decrease in the population of gram-negative bacteria (log_10_CFU/g), such as *E. coli* and gram-positive, such as *S. aureus* in the intestinal content of broiler chickens because of the effect of 17.5 ppm and 35 ppm of PaP and PaF concurs with the results of the minimum inhibitory concentration (MIC) and the minimum bactericidal concentration (MBC) from our previous study. In this study, 6.25–25 mg/mL of five fractions from the *P. aduncum* ethanol extract inhibited the growth and demonstrated *in vitro* bactericidal action against *E. coli* ATCC 25922, *S. aureus* ATCC 25923, *S. typhimurium* ATCC 14028, and *B. subtilis* ATCC 6633.

Although previous studies have revealed that the intestinal microbiota in chickens primarily comprise gram-positive microorganisms (91), phytochemical compounds generally have greater antimicrobial activity against gram-positive bacteria than against gram-negative (88, 92, 93). Gram-negative bacteria release greater levels of endotoxins than gram-positive bacteria. These induce inflammation of the epithelium in the intestinal mucosa, affecting intestinal health to a greater extent.

The reduction in the *E. coli* and *S. aureus* population as log_10_Colony Forming units (log_10_CFU) in the intestinal microbiota of broiler chickens because of PaP and PaF in this study highlights the wide spectrum of antimicrobial activity of these phytocompounds. Similar results are non-existent, however, a reduction in the *E. coli* population in the intestinal contents of chickens has been observed in essential oils or phytogenic additives from other plants (51, 52, 94–96).

Polyphenols are phytocompounds that are found in edible vegetables, nutraceutical, and medicinal plants. They have been extensively studied and have received greater attention because of their antimicrobial activity for a wide range of microorganisms and the gastrointestinal microbiota (27, 28, 36). The reduction in the abundance of these groups of bacteria in the intestinal microbiota of chickens at 21 d of age in this study is aligned with the results obtained in mice, with polyphenols from tea (64) and itcould be associated with the phytocompounds from *P. aduncum* possessing antimicrobial action mechanisms. Asaricin, elemicin and myristicin have been identified as propenylphenols in *Piper aduncum* (25, 57). Previous studies with these three phytocompounds obtained from *Piper sarmentosum* and *P. rivinoides, respectively,* have revealed antimicrobial activity (97, 98). This antimicrobial activity would be associated with the chemical structure that characterizes these phytocompounds (37, 99). Antimicrobial mechanisms of phenols are linked to the hydrophobicity of the molecules, which enter the single membrane, thus disrupting permeability and homeostasis, resulting in a consequent loss of the cellular components and eventual cell death (88, 92, 93).

Nonetheless, PaP and PaF in this study also demonstrated an enhanced effect on the growth of the intestinal microbiota in chickens. It reveals an increase in the abundance of *Lactobacillus* sp. at 21 d of age, in comparison to group C1. This maintains a relationship with what was found in previous studies, wherein some polyphenols have proven to have beneficial influence on the beneficial bacteria in the microbiota, such as *Bifidobacterium* and *Lactobacillus* (27, 64, 99, 100). This highlights the variation in the ability of PaP and the compounds of these polyphenols to modify the intestinal microbiota of chickens, thereby strengthening their intestinal health. To the best of our knowledge, this is the first report on *in vivo* antimicrobial activity of polyphenols and flavonoids from *P. aduncum*.

### 4.4 Intestinal histomorphometry

Previous studies on the effects of polyphenols on gut histomorphometry are not found. However, previous studies have demonstrated that the height of intestinal villi increases because of extracts or essential oils from other plants (13, 15, 101–103). This facilitates the mechanisms for nutrient absorption as it does the mechanisms of the antibiotic as growth promoters, which also promotes an increase in the height of the intestinal villi (104).

The increase in the height and width of the intestinal villi with age, because of the effect of PaP and PaF in this study, can be elucidated by the two principal pharmacological activities recognized for polyphenols: the vast range of antimicrobial activities possessed by these phytocompounds (37, 99) and obtained in this study, as well as its antioxidant activity, observed in previous studies (30–33). This antioxidant activity is consistent with that obtained in a previous result of this study, where the ethanol extract from *P. aduncum* leaves inhibited the DPPH radicals at 65%, and this same extract proved to have a IC_50_ of 98.25 μg/mL. Another marker of the antioxidant activity of these fractions of *P. aduncum* in this study was the decrease in alanine transferase (ALT) profile at 21 d of age in chicken (Table 4). This enzyme is released by tissue cells mainly by hepatocytes and it increases when cells are injured or death (105), conversely it decreases when cells are enhanced by membrane protective chemicals as antioxidants do. The results of this study are aligned with those obtained in a previous study, wherein an increase in the depth of the Lieberkühn crypts and an increase in the width of the villi in relation to the age of the chickens under the effect of supplementing with 0.01% ethanol extract from *P. aduncum* leaves in the diets was reported. The increase in the width adopted a quadratic tendency (23), as well as other previous studies, where the modulation of the intestinal histomorphometry was obtained using sunflower flour, propolis extract, and integral propolis from bees (106–109).

The increase in the structures of the intestinal mucosa, such as the height and width of the villi, increases nutrient absorption and enzyme production owing to more dynamic replacement mechanisms from the enterocytes in the villi. This increase also promotes mechanisms that increase the population of goblet cells, which secrete mucus, and Paneth cells present in birds (110). Villi also secrete antimicrobial products, such as lysozymes and enteroendocrine cells, which secrete local hormones (111, 112) in a balanced manner and contribute to strengthening intestinal health.

### 4.5 Performance indices

Previous studies on the effects of PaP on weight gain, feed intake, and feed conversion rate in broiler chickens have not been conducted yet. Increasing carcass yield, maintaining weight gain and feed conversion rate, reducing feed intake overall in the three stages, in comparison to the chickens from groups C1 and C2, are consistent with the strengthening of gut health influenced by the results of the microbiota modulation, the increase in the height and width of the gut villi of the chickens supplemented with PaP and PaF in this study.

These results are consistent from previous studies using polyphenols from other plants, such as curcumin, resveratrol, and epigallocatechin gallate in broiler chickens, laying hens and quails, where an increase in the weight gain, a decrease in the feed conversion rate, an increase in the production and quality of eggs using mechanisms inherent to their antioxidant activity, have been reported (39–42).

Reduction of feed intake in the chickens supplemented PaP in the present study could be related to the fact that it contains phytochemical compounds which cause similar effects to those containing some phytogenic additives used in chicken diets which causes a modulation of anorexigenic hypothalamic neuropeptides responsible for higher feed efficiency by feed intake reduction and maintaining body weight (113).

At the same time, improvement in the feed efficiency in broilers chicken might be related to the anti-inflammatory effect of PaP obtained in the present study, and in this case at the hepatic tissue level, improving the hepatic metabolism interfering with the accumulation of lipids which are produced from high calorie diets during the fattening phase in broilers chicken (18, 114).

Even though polyphenols, in general, are well known for modulating the intestinal microbiota through their antimicrobial activity mechanisms (36–38), as well as for promoting the health of cells and tissues through their antioxidant activity (29, 34, 35), other studies have identified polyphenols because reduce activity on cellular metabolism through the inhibition of the lipase and cholesterol esterase activity (77, 81, 82), and it also by means of the inhibition of the amylase and glucosidase activity (115, 116).

Herein, inhibition mechanism of these enzymes because of the effect of PaP could primarily elucidate the reduction in triglyceride’s profiles in this study. This could have contributed, consequently, to maintaining weight gain and feed conversion rate and improving carcass yield overall in the three stages in the present study (Table 7). In general, beneficial effects of polyphenols depend on many factors, including plant species, their type, concentration, combination with other compounds, absorption and metabolic transformation, and the cell type or experimental animal species (29, 64, 117).

The results obtained in the present study support the grow promotor activity of PaP and PaF and therefore to be potentially used as an alternative of antibiotics as growth promotors in chickens. However, limitations associated with the practical application of plant extracts and essential oils in poultry arises (1) In general, variations in composition, relative proportions of bioactive phytocomponents as polyphenols in plants depend on factors, such as species, genetics (variety and clones), and environmental factors, such as exposure to light, temperature, and soil. This includes farming practices, such as irrigation, nitrogen availability, and diverse management parameters for farming (64). (2) The supplementation of phytocompounds in feed have some difficulties for the phytocompounds to homogenate, degradation in the feeders and low speed of being absorbed by the gut because of the very small quantities to be used (3) The supplementation in drinking water is easier for the phytocompounds to homogenate, fast in being absorbed by the gut but very difficult to manage the supplementation by itself. Nonetheless, further studies using physio pathological models and molecular techniques are essential to elucidate these nutraceutical effects of polyphenols from *Piper aduncum.*

## 5 Conclusion

Supplementation with 17.5 and 35 ppm of PaP and PaFdecreased the abundance of *Staphylococcus aureus* and *Escherichia coli* in the intestinal microbiota and increased *Lactobacillus* sp., villi length and width in the intestinal mucosa of broiler chickens, indicating improvement of intestinal health. In addition, 17.5 and 35 ppm of PaP and PaF supplementation increased lymphocytes, globulins and reduced ALT and granulocytes. Furthemore, PaP and PaF improve carcass yield, maintain weight gain and feed conversion rate and reduce feed intake overall in the three stages of broiler chickens. This study also showed that PaP and PaF did not have a detrimental effect on any of the parameters evaluated, therefore these phytocompounds enhanced gut health, immunity, anti-inflammatory activity and the performance indices in broiler chicken. In the short term, these results can be used in practical applications, such as (1) improving gut health by modulating the intestinal microbiota and enhancing intestinal villi growth, (2) enhancing immune and anti-inflammatory responses (3) reducing deposition of fat in carcass and obesity (4) using as growth promotors for replacing conventional antibiotics. However, more studies are essential to determine the type of phenols and flavonoids compounds in PaP and PaF and the mechanisms by which these phytocompounds promote these beneficial effects in physio pathological models.

## Data Availability

The original contributions presented in the study are included in the article/supplementary material, further inquiries can be directed to the corresponding author.
